# From Mild Encephalitis Hypothesis to Autoimmune Psychosis - and remaining challenges

**DOI:** 10.1192/j.eurpsy.2022.1797

**Published:** 2022-09-01

**Authors:** K. Bechter

**Affiliations:** University Clinic Ulm, Psychiatry And Psychotherapy 2, Bezirkskrankenhaus Günzburg, Günzburg, Germany

**Keywords:** Mild encephalitis, autoimmune psychosis, immune therapies, neuroinflammation

## Abstract

**Introduction:**

The mild encephalitis (ME) hypothesis of severe mental disorders, ME to be caused by infections, autoimmunity , toxicity and trauma ( Bechter 2001, updated Bechter 2013), is now emergingly supported from neuroimaging and CSF and postmorten findings.

**Objectives:**

Review about the present status of ME hypothesis and autoimmune psychosis and remainig challenges to assess and categorize mild neuroinflammation.

**Methods:**

expert review

**Results:**

Autoimmune Encephalitis presenting with exclusive psychiatric symptoms and all cases of Autoimmune Psychosis (international consensus criteria in Pollak et al, Lancet Psychiatry, 2020) match the proposed ME criteria ( Bechter 2001 & 2013). Majority of these cases of an autoimmune type of ME are well treatable with immune modulatory treatments. It remained unclear, whether CNS antibodies are causal or contributive by shaping the observed clinical syndrome. The increasing evidence of mild neuroinflammation present in considerable subgroup of schizophrenia or psychosis spectrum cases from ongoing clinical studies, including CSF ( Bechter et al 2010, Endres et al 2018, 2020, aso.) and neuroimaging plus the observed clinical improvement with immune modulytory therapies, strongly support ME hypothesis, potentialy even in considerably larger subgroup of SMDs,supported by brain biopsy (Najjar et al ,several papers) and post mortem studies (Bogerts et al group ,Weickert et al group, several papers).

**Conclusions:**

Beyond ME even more refined categories of mild neuroinflammation, including parainflammation ( proposed by Medzhitov 2008) and neuroprogression ( proposed by Berk et al 2010/11) need to be considered in further research on the possible role of mild neuroinflammation in SMDs (Bechter 2020, Frontiers Psychiatry).

**Disclosure:**

No significant relationships.

